# *CHES-1-like*, the ortholog of a non-obstructive azoospermia-associated gene, blocks germline stem cell differentiation by upregulating Dpp expression in *Drosophila* testis

**DOI:** 10.18632/oncotarget.9789

**Published:** 2016-06-02

**Authors:** Jun Yu, Yujuan Liu, Xiang Lan, Hao Wu, Yang Wen, Zuomin Zhou, Zhibin Hu, Jiahao Sha, Xuejiang Guo, Chao Tong

**Affiliations:** ^1^ State Key Laboratory of Reproductive Medicine, Nanjing Medical University, Nanjing 211166, China; ^2^ Life Sciences Institute and Innovation Center for Cell Signaling Network, Zhejiang University, Hangzhou 310058, China; ^3^ Department of Histology and Embryology, Nanjing Medical University, Nanjing 211166, China; ^4^ Animal Core Facility, Nanjing Medical University, Nanjing 211166, China

**Keywords:** non-obstructive azoospermia, Drosophila, testis tumor, BMP signaling, germline stem cells

## Abstract

Azoospermia is a high risk factor for testicular germ cell tumors, whose underlying molecular mechanisms remain unknown. In a genome-wide association study to identify novel loci associated with human non-obstructive azoospermia (NOA), we uncovered a single nucleotide polymorphism (rs1887102, P=2.60 ×10^−7^) in a human gene *FOXN3. FOXN3* is an evolutionarily conserved gene. We used *Drosophila melanogaster* as a model system to test whether *CHES-1-like*, the *Drosophila FOXN3* ortholog, is required for male fertility. *CHES-1-like* knockout flies are viable and fertile, and show no defects in spermatogenesis. However, ectopic expression of CHES-1-like in germ cells significantly reduced male fertility. With CHES-1-like overexpression, spermatogonia fail to differentiate after four rounds of mitotic division, but continue to divide to form tumor like structures. In these testes, expression levels of differentiation factor, Bam, were reduced, but the expression region of Bam was expanded. Further reduced Bam expression in CHES-1-like expressing testes exhibited enhanced tumor-like structure formation. The expression of *daughters against dpp* (*dad*), a downstream gene of dpp signaling, was upregulated by CHES-1-like expression in testes. We found that CHES-1-like could directly bind to the *dpp* promoter. We propose a model that CHES-1-like overexpression in germ cells activates *dpp* expression, inhibits spermatocyte differentiation, and finally leads to germ cell tumors.

## INTRODUCTION

Testicular germ cell tumors (TGCTs) are the most common cancer among young men in industrialized countries [1]. TGCTs are thought to be derived from germ cell lineage cells that are blocked in differentiation and maturation [1]. The causative genetic aberrations were rarely identified. Genome-wide association study (GWAS) studies revealed polymorphic loci linked to the KIT/KITLG [2, 3], RAS [4], and steroid signaling pathways [5, 6]. However, the molecular mechanisms underlining TGCTs remain poorly understood.

*Drosophila* and human testes share many features of spermatogenesis [7, 8]. Many mutants of homologous human and *Drosophila* genes exhibit similar testicular phenotypes. The adult fly testis is a blind tube that opens into the seminal vesicle and ejaculatory duct [8]. The apical tip of the tube is a cluster of somatic cells called hub cells. Eight to ten germ line stem cells (GSCs) are tightly associated with the hub cells, and each is enveloped by two cyst-stem cells (CySCs). Each GSC divides asymmetrically to maintain one cell associated with the hub as a GSC, and another to leave the niche and become a primary spermatogonial cell. Spermatogonial cells undergo four rounds mitosis before further differentiation, and then enter meiosis and mature into spermatids [9].

The self-renewal and differentiation of early germ cells in flies are tightly controlled [9]. Similar to humans, flies also develop testis tumors when germ cells fail to differentiate and over-proliferate [10]. Janus kinase-signal transducer and activator of transcription (JAK-STAT) and bone morphogenetic protein (BMP) signaling are critical for GSC maintenance [8, 9]. Malfunction of these two pathways could lead to testis tumors in flies. Hub cells secrete Unpaired (Upd) to bind receptor Dormless on GSCs and CySCs, which activates JAK-STAT signaling and maintains germline and somatic stem cell self-renewal [11, 12]. The ectopic expression of Upd in GSCs results in testis tumors with a massive accumulation of undifferentiated GSC-like cells [11, 12].

Two BMP-like molecules, Dpp and Gbb, expressed in hub and cyst cells are required for GSC maintenance [13–15]. Dpp and Gbb are received by GSCs, where they repress the expression of the differentiation factor, Bag-of-marbles (Bam) [13–15]. Bam and its regulator, Benign gonial cell neoplasm (Bgcn), are required for restricting proliferation of mitotically amplifying spermatogonia [16, 17]. Mutations in *bam* or *bgcn* lead to testis tumors with extensive proliferation of undifferentiated germ cells [18, 19]. Since BMP signaling could repress *bam* expression, ectopic expression of *dpp* in germ cells leads to reduced *bam* expression and the formation of tumor-like structures in testis [13, 15]. Despite its important functions in fly spermatogenesis, BMP signaling is also required in testis development and spermatogenesis in mammalian systems [20]. Aberrant BMP signaling was reported in human samples with TGCTs [21]. Therefore, investigation of germ cell differentiation in flies might provide insight into potential mechanisms for human TGCTs.

Our previous work has successfully used *Drosophila* testis as a model system to evaluate the possible loci associated with a severe symptom of male infertility: non-obstructive azoospermia (NOA) [22, 23]. We found two loci near *DMRT2* and *DMRT3*, two genes encoding a DM domain containing a transcription factor, were associated with NOA [22]. Interestingly, *DMRT1*, the paralog of *DMRT2/3*, was found to be associated with human TGCTs [24, 25]. Studies showed that the risk of TGCTs was increased in azoospermia or subfertile patients [1, 26–28]. Therefore, genes associated with azoospermia might also modulate the risk of TGCTs.

In the same GWAS study [23, 29], we uncovered that a single nucleotide polymorphism (SNP) in the human *FOXN3* gene is associated with NOA. FOXN3 is evolutionary conserved. As indicated in Ensembl database, fly gene *CHES-1-like* is the ortholog of both human *FOXN3* and *FOXN2*, which is a one to multiple orthologous relationship. To evaluate the functional relevance of the GWAS study, we assessed the function of *CHES-1-like* in fly spermatogenesis. *CHES-1-like* mutant male flies were viable and fertile. We found that *CHES-1-like* is not required for GSC maintenance or other spermatogenesis processes in fly testes. However, ectopic expression of *CHES-1-like* in germ cells significantly reduced male fertility. When *CHES-1-like* was overexpressed, spermatogonia failed to differentiate after four rounds of mitotic division, but continued to divide to form tumor-like structures. We found that *CHES-1-like* could activate *dpp* expression and block spermatocyte differentiation. Our results suggest that NOA-associated SNPs could be a potential modulator of testis tumor development.

## RESULTS

### Loss of *CHES-1-like* does not cause spermatogenesis defects

**Figure 1:**
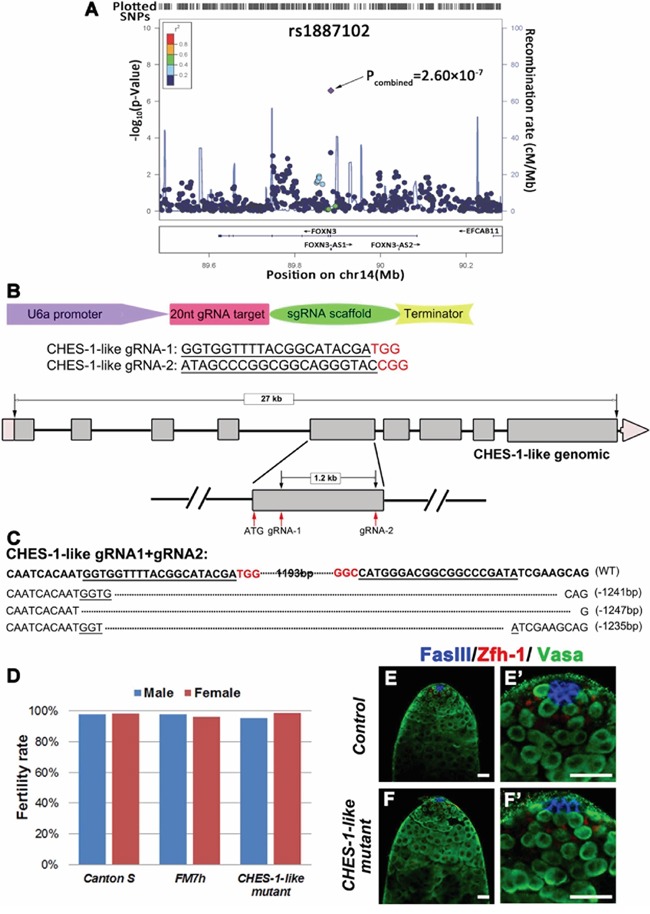
Loss of *CHES-1-like* does not cause spermatogenesis defects in *Drosophila* **A.** The SNP rs1887102 at 14q32.11 was identified as a NOA associated SNP. Additive models of logistic regression analyses were used to estimate the P-value of association analyses in 981 NOA cases and 1,657 controls. The marker SNP is shown in purple and the r^2^ value of the rest of the SNPs are indicated by different colors. The genes within the region of interest are annotated, with arrows indicating transcription direction. **B.** The design of knock out (KO) strategy for *CHES-1-like* gene using CRISPR/Cas9 technology. The scheme shows that the design of the injected construct, sequence of gRNAs, and the location of the gRNAs. **C.** Indels identified in Three *CHES-1-like* alleles. **D.** The fertility of controls and *CHES-1-like* mutants. **E-F'.** There is no structural defects of the *CHES-1-like* KO testes. FasIII labels hub cells (Blue), Zfh-1 labels cyst stem cells (Red), Vasa labels germ cells (Green). (E') and (F') are enlarged images of (E) and (F).

In our previous NOA GWAS screen [23, 29], one SNP (rs1887102, P=2.60 ×10^−7^) in the human gene, *FOXN3*, was found to be associated with NOA (Figure 1A). *FOXN3* is an evolutionarily conserved gene. In *Drosophila, CHES-1-like* is the ortholog of *FOXN3*. To evaluate whether this loci is functionally relevant to spermatogenesis, we tested the function of *CHES-1-like* in fly testes.

We knockdown *CHES-1-like* expression in germ cells of fly testes (*Nos>CHES-1-like RNAi*) and did not observe obvious defects (Supplementary Figure 1). We generated *CHES-1-like* deletion mutants using Cas9-mediated mutagenesis. We recovered multiple lines with different indels confirmed by PCR and sequencing (Figure 1B, 1C). Both the hemizygous mutant male flies and homozygous mutant female flies were viable and fertile (Figure 1D). We further examined the testes of *CHES-1-like* mutants by immunostaining with antibodies recognizing hub cells, germ cells, and cyst cells (Figure 1E, 1F). The patterns of all cell types were identical to the wild type controls, indicating that loss of *CHES-1-like* does not affect spermatogenesis in flies.

### Ectopic expression of *CHES-1-like* in germ cells induces testis tumor formation

**Figure 2:**
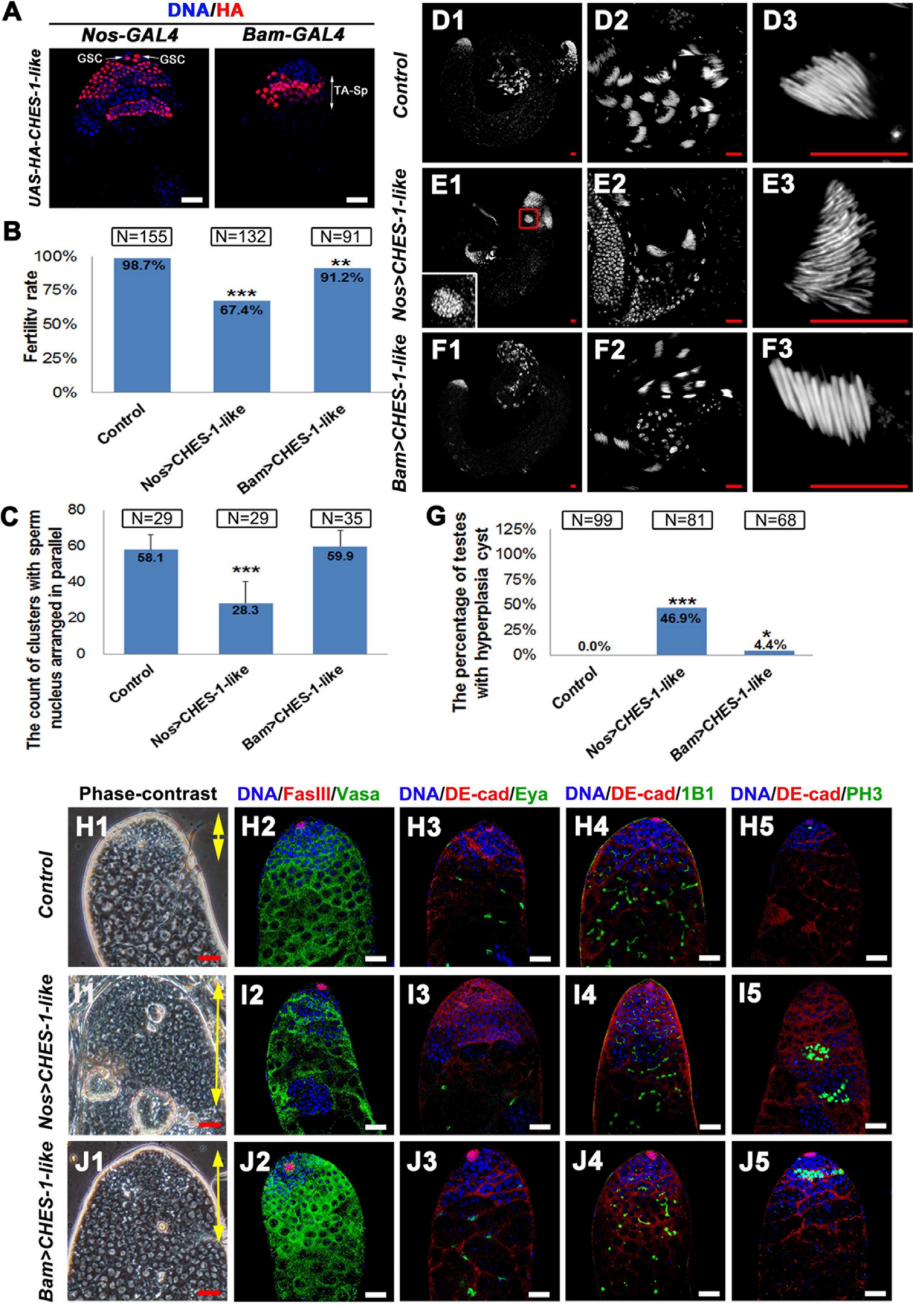
Ectopic expression of CHES-1-like in germ cells causes male fertility defect and tumor like structure formation **A.** The patterns of *Nos-Gal4* and *Bam-Gal4* driving HA tagged CHES-1-like protein expression. **B.** The fertility rate of male flies with indicated genotypes. CHES-1-like overexpression in germ cells reduced male fertility. Chi-square test was used. ***: P<0.001; **: P<0.01. **C.** The quantification of the clustered elongated spermatids at the proximal ends of the testes with indicated genotypes. *Nos>CHES-1-like* testes have reduced numbers of elongated spermatids. Student's t test was used, ***: P<0.001. **D-F.** Whole mount testes with indicated genotypes were stained with Hochest 33342. (D2-F2 and D3-F3) are enlarged views of (D1-F1) to show the proximal end of the testes and clustered spermatids. The boxed region of (E1) shows a cyst with over-proliferated germ cells. **G.** The quantification of testes containing cysts with over-proliferated germ cells (hyperplasia cysts). Chi-square test was used. ***: P<0.001; *: P<0.05. **H1-J1.** The phase contrast images of testes with indicated geneotypes show an increasing of the small cells that resembled GSCs and spermatognia in the apical region of both *Nos>CHES-1-like* and *Bam>CHES-1-like* testes (areas indicated by yellow double head arrows). The hyperplasia cysts were indicated by red arrows. (H2-J5) Immunostaining of testes with indicated geneotypes. DNA, germ cells, hub cells, cyst cells, mature cyst cells, fusomes, and dividing cells were labeled with Hochest 33342, Vasa, FasIII, DE-Cadherin, Eya, 1B1, and pH3, respectively. *Nos>CHES-1-like* testes has overproliferated spermatogonial cells indicated by small branched fusomes and pH3 labeling. Scale bars: 20 μM.

Since *CHES-1-like* loss of function did not result in spermatogenesis defects, we decided to examine whether ectopic expression of *CHES-1-like* could lead to testis malfunction. We generated *UAS-CHES-1-like* transgenic flies, and crossed these flies with different Gal4 lines expressing specifically in germ cells (*Nos-Gal4* and *Bam-Gal4*; Figure 2A), cyst cells (*Tj-Gal4*; Supplementary Figure 2), and hub cells (*Upd-Gal4*; Supplementary Figure 3) in fly testes.

Fertility was significantly reduced in *Nos>CHES-1-like* (N=132) and *Bam>CHES-1-like* (N=91) male flies (Figure 2B). DNA staining in the testis tail revealed that clusters of elongated spermatids were greatly reduced in *Nos>CHES-1-like* testes compared to normal testes (Figure 2C–2F). An increase in the small cells resembling GSCs and spermatogonia in the apical region of both *Nos>CHES-1-like* and *Bam>CHES-1-like testes* was observed by phase contrast microscopy (Figure 2H1, 2I1, 2J1). Cysts with an overproliferation of small cells were also observed in the distal region of the *Nos>CHES-1-like* testis tips (Figure 2G, 2H1, 2I1, 2J1).

We further examined *Nos>CHES-1-like* and *Bam>CHES-1-like* testes with various cellular markers (Figure 2H–2J). DNA and Vasa staining confirmed that the region of GSCs and spermatogonial cells were expanded (Figure 2H2, 2I2, 2J2). In the *Nos>CHES-1-like* testes, cysts packed with small cells form tumor-like structures in which Vasa staining is lost (Figure 2I2). We used the 1B1 antibody to label fusome, a membrane structure that connects sibling germ cells. Most fusomes observed in the apical tip of *Nos>CHES-1-like* and *Bam>CHES-1-like* testes were small and branched (Figure 2H4, 2I4, 2J4), suggesting that the majority of cells were interconnected and resembled proliferating spermatogonia. This indicates that the spermatogonial cells failed to cease mitotic division after four rounds, and continually divided to form tumor-like structures. Indeed, using the cell proliferation marker phospho-Histone H3 (pH3) antibody to label dividing germ cells, pH3 could be observed only at the tip of wild type testes (Figure 2H5). However, ectopic pH3 labeling was observed in some cysts distal from the tip of the testes with ectopic *CHES-1-like* expression in germ cells (Figure 2I5, 2J5). We also used FasIII to label hub cells, and Eya and DE-cadherin to label cyst cells. There was no dramatic change in the number and morphology of either cell type (Figure 2H–2J and Supplemental Figure 1).

We further investigated Tj-Gal4-driven *CHES-1-like* expression in cyst cells and Upd-Gal4-driven *CHES-1-like* expressionin hub cells, and examined the resulting testes with various markers. There was no obvious difference between wild type testes and the testes with *CHES-1-like* expression in cyst (Supplementary Figure 2) and hub cells (Supplementary Figure 3).

### *CHES-1-like* inhibits spermatocyte differentiation through suppressing Bam expression

**Figure 3:**
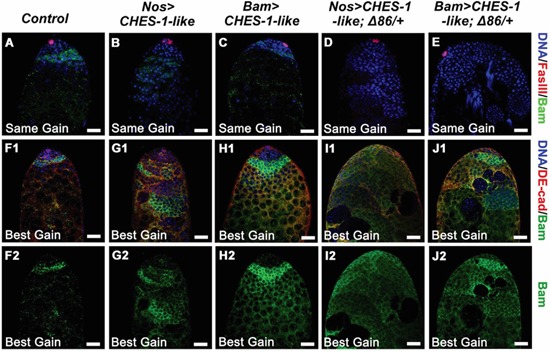
Ectopic expression of CHES-1-like in germ cells led to reduced level of Bam expression **A-E.** Bam expression levels are greatly reduced in the testes with CHES-1-like ectopic expression in germ cells. The same gain were used to indicate the level of Bam expression, DNA (Blue), hub cells (Red), and Bam (Green) were labeled with Hochest 33324, FasIII, and Bam antibodies. Remove one copy of *bam* (*Δ86/+*) further decreased Bam expression. **F1-J2.** Using longer exposure time (best gain) to show the signals of Bam staining, expended Bam expression regions were observed in the testes with CHES-1-like overexpressed in germ cells. DE-cadherin (Red in F1-J1) labels cyst cells. (F2-J2) are the green channels of (F1-J1). Scale bars: 20 μM.

The expression of Bam is required for spermatogonial cells to exit the mitotic cell cycle and begin differentiation [19]. It is likely due to the disruption of Bam signaling that spermatogonia were unable to cease mitosis in CHES-1-like germ cell ectopically expressing testes. To test this, we analyzed Bam expression patterns in these testes. In wild type animals, Bam is expressed primarily in the transient amplifying (TA) spermatogonia, a strip of cells near the apical tip of the testes (Figure 3A). The expression level of Bam was greatly reduced in both *Nos>CHES-1-like* (Figure 3B) and *Bam>CHES-1-like* testes (Figure 3C). However, the expression region of Bam was expanded (Figure 3F1–3J1, 3F2–3J2). We also used Bam-GFP reporter to analyze *bam* expression patterns. In *Nos>CHES-1-like* testes, GFP expression levels were reduced and the expression regions were expanded (Supplementary Figure 4). The reduction of GFP staining is obvious but less dramatic than the reduction of Bam staining in *Nos>CHES-1-like* testis (Figure 3B, Supplementary Figure 4), which is likely due to that not only the transcriptional levels but also the protein levels of Bam is affected by CHES-1-like expression.

**Figure 4:**
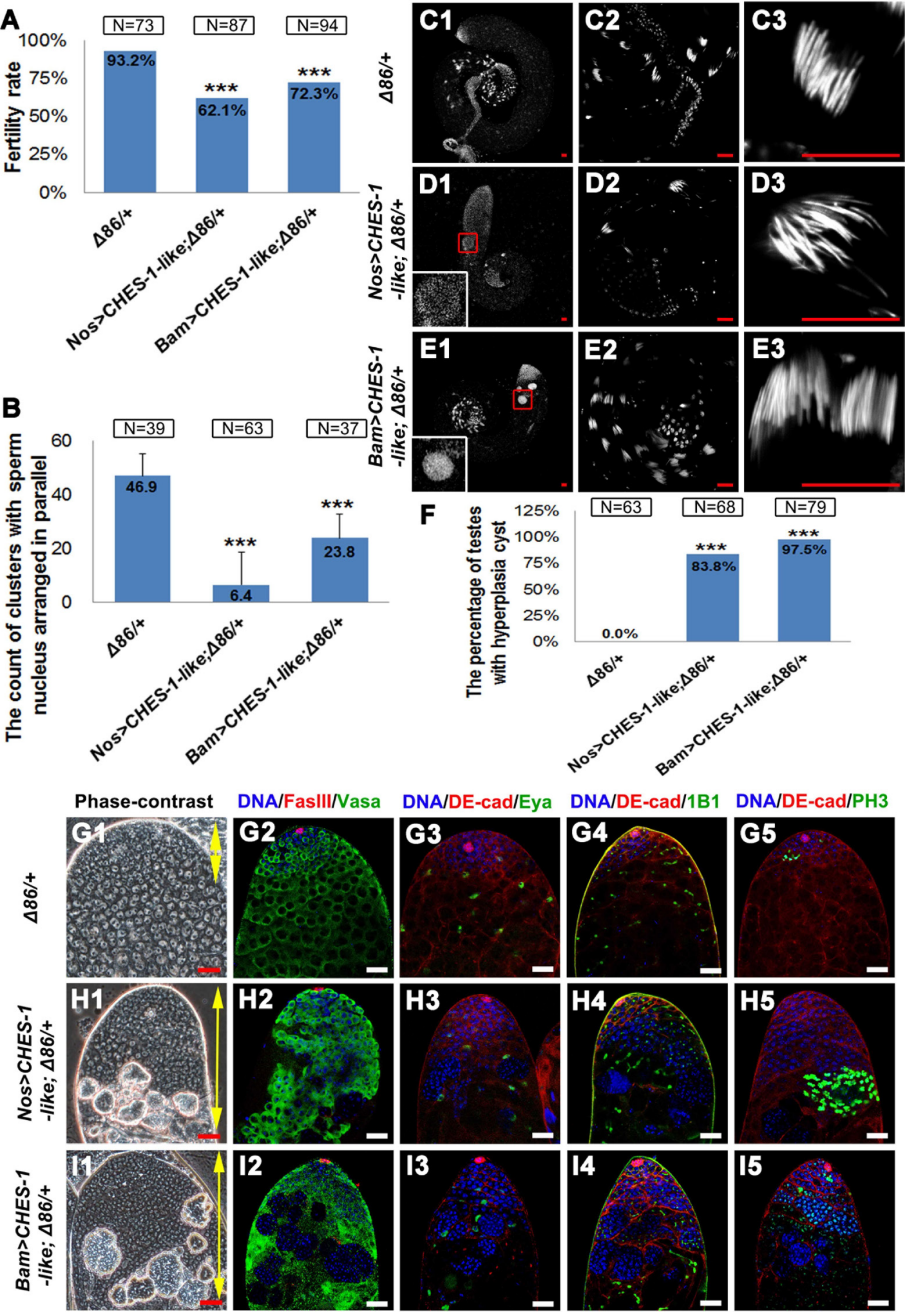
Removing one copy of bam enhanced CHES-1-like overexpression phenotypes **A.** Male fertility rates are further reduced in the *Nos>CHES-1-like; Δ86/+* and *Bam>CHES-1-like; Δ86/+* animals. Chi-square test was used. ***: P<0.001. **B.** The quantification of the clustered elongated spermatids at the proximal ends of the testes with indicated genotypes. Removing one copy of *bam* further reduced the numbers of elongated spermatids in CHES-1-like overexpression testes. Student's t test was used, ***: P<0.001. **C-E.** Whole mount testes with indicated genotypes were stained with Hochest 33342. (C2-E2 and C3-E3) are enlarged views of (C1-E1) to show the proximal end of the testes and clustered spermatids. The boxed regions of (D1 and E1) show cysts with over-proliferated germ cells. **F.** The quantification of testes containing cysts with over-proliferated germ cells (hyperplasia cysts). Chi-square test was used. ***: P<0.001. **G-I.** The phase-contrast views and the immunostaining views of the apical tips of testes with indicated genotypes. Loss of one copy of Bam alone does not cause dramatic defects in testes. Removing one copy of *bam* in Nos>CHES-1-like and Bam>CHES-1-like tests greatly enhanced CHES-1-like overexpressing phenotypes. DNA, germ cells, hub cells, cyst cells, mature cyst cells, fusomes and dividing cells were labeled with Hochest 33342, Vasa, FasIII, DE-Cadherin, Eya, 1B1, and PH3, respectively.

It has been reported that around half of male germ cells lacking one copy of *bam* will complete one or more extra TA divisions before differentiation [30]. Removing one copy of *bam* further reduced the fertility of male flies with CHES-1-like overexpressing in germ cells (Figure 4A). DNA staining in the testis tail revealed that clusters of elongated spermatids were further reduced in these testes compared to the testes with CHES-1-like overexpressing alone (Figure 4B and 4C1–4E3). Bam expression in these testes was reduced to an undetectable level (Figure 3D, 3E). There was an increase in aberrant tumor-like cysts packed with over-proliferated small cells (Figure 4F–4I). These results suggest that CHES-1-like likely suppresses spermatocyte differentiation by down-regulating Bam expression.

### *CHES-1-like* activates TGF-β signaling by upregulating *dpp* expression

**Figure 5:**
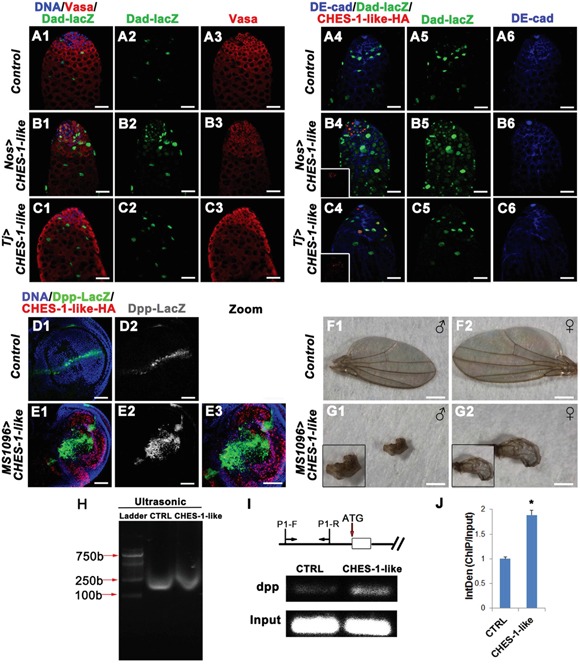
CHES-1-like prevents spermatogonial differentiation through promoting Dpp expression to ectopically activate BMP signaling in germ cells **A1-C6.** Over-expressing CHES-1-like in germ cells (*Nos>CHES-1-like)* but not cyst cells (*Tj>CHES-1-like*) activates BMP down-stream gene *dad* reporter *dad-lacZ* expression in germ cells. DNA, germ cells, cyst cells, dad-lacZ, and HA tagged CHES-1-like were labeled with Hochest 33342, Vasa, DE-Cadherin, LacZ, and HA anitibodies. **D1-E3.** CHES-1-like overexpression in wing imaginal discs ectopically induces *dpp* expression in wing porch. Dpp expression was monitored by reporter *dpp-lacZ*. **F1-G2.** Images of adult wings of animals with indicated genotypes. **H-J.** ChIP assay indicates that CHES-1-like could bind to *dpp* promoter region. (H) Chromatin from control (CTRL) and HA-CHES-1-like transfected S2 cells was sonicated to obtain DNA fragments of appropriate size among 100-1000 bp. (I) Scheme shows the genomic region of dpp gene and the primers (P1-F, P1-R) used for CHIP assay. HA antibody could pull down *dpp* promoter region. (J) Quantification of (I).

In testis, Bam expression is repressed by BMP signaling. Therefore, CHES-1-like-mediated down regulation of Bam expression might reflect the activation of BMP signaling. Indeed, the BMP signaling downstream gene *daughters against dpp* (*dad*) was expressed at low levels in GSCs and spermatogonial cells in wild type testes (Figure 5A1–5A6). Expression levels of Dad-lacZ dramatically increased in the germ cells expressing CHES-1-like (Figure 5B1–5B6). However, ectopic expression of CHES-1-like in early cyst cells with Tj-Gal4 did not increase Dad-lacZ expression in cyst cells (Figure 5C1–5C6). The upregulation of Dad expression and downregulation of Bam expression indicates that CHES-1-like may ectopically activate BMP signaling.

BMP downstream gene expression is mediated by phosphor-Mad (p-Mad) and its cofactor, Medea. Since CHES-1-like is a FOX domain-containing protein, a potential transcriptional factor, we first tested whether it could interact with Mad to regulate Dad and Bam expression. However, we did not detect any interaction between CHES-1-like and Mad (Supplementary Figure 5).

Because ectopic expression of CHES-1-like in germ cells mimicked the phenotype of Dpp overexpression in germ cells [13, 15], we tested whether CHES-1-like could directly upregulate *dpp* expression. We used *MS1096-Gal4*-driven *CHES-1-like* expression in the wing imaginal disc porches and examined Dpp-lacZ expression patterns (Figure 5D–5G). In the wild type wing discs, Dpp-lacZ appeared as a thin strip at the anterior/posterior (A/P) boundary (Figure 5D1, 5D2). When CHES-1-like was overexpressed, the expression region of Dpp-lacZ was greatly expanded (Figure 5E1–5E3). As a result, the adult wings of the *MS1096>CHES-1-like* flies were deformed (Figure 5F1–5G2). However, another gene *ptc* does not change its expression patterns in the wing porches when CHES-1-like is overexpressed (Supplementary Figure 6), indicating that the changes of Dpp-LacZ expression patterns is not due to the morphological changes of the wing discs.

To test whether CHES-1-like directly binds to the *dpp* promoter to activate Dpp expression, we used a chromatin immunoprecipitation (ChIP) assay to detect the interaction between CHES-1-like and the *dpp* promoter. We expressed HA-tagged CHES-1-like in *Drosophila* S2 cells, and immunoprecipitated the protein-DNA complex after cross-linking. Indeed, CHES-1-like was able to interact with the *dpp* promoter region (Figure 5H–5J).

## DISCUSSION

In this study, we used *Drosophila* testis as a model system to test the functional relevance of a NOA-associated human gene, *FOXN3*. Although loss of *CHES-1-like*, the fly ortholog of *FOXN3*, did not result in spermatogenesis defects, the ectopic expression of *CHES-1-like* in germ cells blocked spermatocyte differentiation and induced tumor-like structure formation. Our study has revealed CHES-1-like as a novel BMP signaling regulator to promote expression of the BMP ligand Dpp.

In flies, CHES-1-like, together with another fork head transcription factor Jumu, governs cardiac progenitor cell division and specification by regulating Polo kinase activity, as well as the expression of fibroblast growth factor and Wnt signaling pathway receptors [31, 32]. Inactivation of FOXN3 in *Xenopus* and mice led to craniofacial defects and was sometimes lethal [33, 34].

It has been well established that BMP signaling is required for the differentiation and proliferation of osteoblasts of the mammalian skull [35]. Interestingly, the expression levels of the BMP pathway ligands BMP2, BMP4, and BMP7 were greatly reduced in the *FOXN3* knockout animals [34]. In our study, we found that CHES-1-like binds to the *dpp* promoter and activated Dpp expression, suggesting that CHES-1-like might directly regulate BMP ligand expression. Dpp is critical for fly development [36, 37]. However, loss of CHES-1-like did not result in any obvious developmental defect. It is possible that there is redundant molecules or pathways could compensate the effects caused by the loss of *CHES-1-like*. Jumu is a good candidate since it is playing redundant roles during fly heart development. However, ectopic expressing Jumu in germ cells (Nos>UAS-Jumu) does not have any defects (Supplementary Figure 1), indicating Jumu might not play similar roles as CHES-1-like in testes.

The function of FOXN3 has never been linked to testis tumors or male sterility. The data from human protein atlas (http://www.proteinatlas.org/) revealed that the protein levels of FOXN3 were upregulated in most cancer tissues. A recent study in a cancer cell line indicated that CHES1/FOXN3 decreases cell proliferation by repressing PIM2 and protein biosynthesis, suggesting that FOXN3 is a potential tumor suppressor [38]. However, we found that overexpression of CHES-1-like in fly testis could prevent early germ cell differentiation and lead to tumor-like structure formation. This paradox might arise because of the complex and context-dependent physiological functions of BMP signaling [39]. Therefore, whether FOXN3 promotes or inhibits tumor formation might be determined by the tissue context.

In this study, we found that the ortholog of a NOA-associated gene could modulate testis tumor formation. It is striking since infertility is a high risk factor of testis tumors. The misregulation of FOXN3 could contribute to both spermatogenesis defects and tumor genesis. Further investigation is necessary to dissect the role of FOXN3 in TGCTs.

## MATERIALS AND METHODS

### Fly strains

All flies were cultured on standard corn meal food at 25°C. Information for alleles and transgenes used in this study can be found either in FlyBase or as noted: *Nos-Gal4* (BDSC, #4937), *Tj-Gal4* (DGRC, #104055), *Upd-Gal4* (BDSC, #26796), *MS1096-Gal4* (THFC, #TB00038), CHES-1-like RNAi (THFC, #THU2388), *Dpp-lacZ, Dad-lacZ*, BMPGFP/+, and *Bam-Gal4;Δ86/+* are gifts from DH Chen.

### Generation of *CHES-1-like* transgenic fly

*CHES-1-like* CDS (BDGP, clone RE02128) was subcloned into the *pUAS-attB-HA* vector. Primers used for PCR are: forward: 5- AAAAGCGGCCGCAATGTCCACAGATAATCCCACACAG-3 and reverse: 5- GTTCTAGATTACCGCATCCAGGCGGACT-3. *Jumu* CDS (BDGP, clone LD24749) was subcloned into the *pUAS-attB* vector. Primers used for PCR are: forward: 5-ATAAGAATGCGGCCGCGATGTTCGAACTAGAGGATTATTCGA-3 and reverse: 5-CCGCTCGAGCTAGATGACGCGGTTGACCAGC-3. The transgenic flies were generated using standard procedures.

### CRISPR/Cas9-mediated genome editing

*CHES-1-like* mutant was generated by an optimized CRISPR/Cas9-mediated genome editing method as described before [40]. Two sgRNAs targeting *CHES-1-like* exon regions were designed to generate an about 1.2kb DNA fragment deletion. Genomic DNA PCR and sequencing were used to confirm the deletions.

### Light and phase-contrast microscopy

Fly testes were dissected in 1x phosphate-buffered saline (PBS) and washed several times. Testes were observed on slides by a phase-contrast microscope after gently squashing them with a cover slip. For an overall view of wing morphology, adult wings were observed directly under light microscope.

### Immunofluorescence and antibodies

Fly testes were dissected in 1x PBS and fixed for 30 min in 4% paraformaldehyde. After washing three times in 1x PBS with 0.1% Triton X–100 (PBST) and blocking for 1hr in 5% bovine serum albumin (BSA), the samples were incubated with primary antibodies overnight at 4°C. After washing three times for 10 min in 0.1% PBST, the samples were incubated for 1 hr with secondary antibodies at room temperature followed by three times washing in 0.1% PBST. Testes were then stained with Hoechst 33342 (1.0 mg/ml, Invitrogen) for 5 min before mounting. The antibodies used were as follows: mouse anti-Eya (DSHB, 1:20); mouse anti-FasIII (DSHB, 1:50); rat anti-DE-cadherin (DSHB, 1:20); mouse anti-1B1 (DSHB, 1:75); rabbit anti-Bam C (a gift from DH Chen, 1:2000) [41]; rabbit anti-Vasa (1:1000, Santa Cruz); rabbit anti-PH3 (CST, 1:400); mouse anti-lacZ (Promega, 1:1000); rabbit anti-HA-tag (CST, 1:1000), rat anti-Zfh1 (C Tong lab, 1:2000), mouse anti-Ptc (DSHB, 1:100), chicken anti-GFP (Abcam, 1:1000). Secondary antibodies conjugated to A488, Cy3, A594, or A647 (Molecular Probes and Jackson Immunologicals) were diluted at 1:1000.

### Chromatin immunoprecipitation (ChIP) assay

Formaldehyde cross-linking and chromatin immunoprecipitation (ChIP) assays of S2 cells were performed using a protocol as described before [42]. S2 cells transfected with HA-CHES-1-like were subjected for CHIP assay. Chromatin was sonicated on ice to obtain DNA fragments of appropriate size among100-1000 bp. Twenty percent of total supernatant was used as a total input control. Following removal of bound proteins, immunoprecipitated DNA was subjected to PCR.

The primer pair used for the CHES-1-like CHIP assays are: 5-CACACACGCTCAGAGACACA-3 and 5-CAAGCGGGACGACTATAGGG-3.

### Immunoprecipitation (IP)

pUAS-Myc-Mad and pUAS-Flag-Medea were made by inserting PCR products of CDS of Mad and Medea into pUAS-Myc and pUAS-Flag constructs. Following primers were used: Mad-Myc F:5-AATTGCGGCCGCATGGACACCGACGATGTG-3, Mad-Myc R: 5-ACGTCTAGATTAGGATACCGAACTAATTGC-3, Medea-Flag F:5- AATCTCGAGATGGGCGGCGGCTCGGG-3, Medea-Flag R: 5-ATTTCTAGATTAGGCGGCGGCACGCGG-3.S2 cells transfected with Myc-Mad and HA-CHES-1-like or Flag-Medea were subjected to IP assays. IP were performed as described [43].

## SUPPLEMENTARY FIGURES



## References

[1] Meyts ER, McGlynn KA, Okamoto K, Jewett MA, Bokemeyer C (2015). Testicular germ cell tumours. Lancet.

[2] Kanetsky PA, Mitra N, Vardhanabhuti S, Li M, Vaughn DJ, Letrero R, Ciosek SL, Doody DR, Smith LM, Weaver J, Albano A, Chen C, Starr JR, Rader DJ, Godwin AK, Reilly MP (2009). Common variation in KITLG and at 5q31. 3 predisposes to testicular germ cell cancer. Nat Genet.

[3] Rapley EA, Turnbull C, Al Olama AA, Dermitzakis ET, Linger R, Huddart RA, Renwick A, Hughes D, Hines S, Seal S, Morrison J, Nsengimana J, Deloukas P, Rahman N, Bishop DT, Easton DF (2009). A genome-wide association study of testicular germ cell tumor. Nat Genet.

[4] Goddard NC, McIntyre A, Summersgill B, Gilbert D, Kitazawa S, Shipley J (2007). KIT and RAS signalling pathways in testicular germ cell tumours: new data and a review of the literature. Int J Androl.

[5] Ferlin A, Ganz F, Pengo M, Selice R, Frigo AC, Foresta C (2010). Association of testicular germ cell tumor with polymorphisms in estrogen receptor and steroid metabolism genes. Endocr Relat Cancer.

[6] Kristiansen W, Andreassen KE, Karlsson R, Aschim EL, Bremnes RM, Dahl O, Fossa SD, Klepp O, Langberg CW, Solberg A, Tretli S, Adami HO, Wiklund F, Grotmol T, Haugen TB (2012). Gene variations in sex hormone pathways and the risk of testicular germ cell tumour: a case-parent triad study in a Norwegian-Swedish population. Hum Reprod.

[7] Davies EL, Fuller MT (2008). Regulation of self-renewal and differentiation in adult stem cell lineages: lessons from the Drosophila male germ line. Cold Spring Harb Symp Quant Biol.

[8] Fuller MT, Spradling AC (2007). Male and female Drosophila germline stem cells: two versions of immortality. Science.

[9] Spradling A, Fuller MT, Braun RE, Yoshida S (2011). Germline stem cells. Cold Spring Harb Perspect Biol.

[10] Hime GR, Loveland KL, Abud HE (2007). Drosophila spermatogenesis: insights into testicular cancer. Int J Androl.

[11] Kiger AA, Jones DL, Schulz C, Rogers MB, Fuller MT (2001). Stem cell self-renewal specified by JAK-STAT activation in response to a support cell cue. Science.

[12] Tulina N, Matunis E (2001). Control of stem cell self-renewal in Drosophila spermatogenesis by JAK-STAT signaling. Science.

[13] Bunt SM, Hime GR (2004). Ectopic activation of Dpp signalling in the male Drosophila germline inhibits germ cell differentiation. Genesis.

[14] Kawase E, Wong MD, Ding BC, Xie T (2004). Gbb/Bmp signaling is essential for maintaining germline stem cells and for repressing bam transcription in the Drosophila testis. Development.

[15] Shivdasani AA, Ingham PW (2003). Regulation of stem cell maintenance and transit amplifying cell proliferation by tgf-beta signaling in Drosophila spermatogenesis. Curr Biol.

[16] Gonczy P, Matunis E, DiNardo S (1997). bag-of-marbles and benign gonial cell neoplasm act in the germline to restrict proliferation during Drosophila spermatogenesis. Development.

[17] Lavoie CA, Ohlstein B, McKearin DM (1999). Localization and function of Bam protein require the benign gonial cell neoplasm gene product. Dev Biol.

[18] Gateff E (1982). Gonial cell neoplasm of genetic origin affecting both sexes of drosophila melanogaster. Prog Clin Biol Res.

[19] McKearin DM, Spradling AC (1990). bag-of-marbles: a Drosophila gene required to initiate both male and female gametogenesis. Genes Dev.

[20] Young JC, Wakitani S, Loveland KL (2015). TGF-beta superfamily signaling in testis formation and early male germline development. Semin Cell Dev Biol.

[21] Fustino N, Rakheja D, Ateek CS, Neumann JC, Amatruda JF (2011). Bone morphogenetic protein signalling activity distinguishes histological subsets of paediatric germ cell tumours. Int J Androl.

[22] Yu J, Wu H, Wen Y, Liu Y, Zhou T, Ni B, Lin Y, Dong J, Zhou Z, Hu Z, Guo X, Sha J, Tong C (2015). Identification of seven genes essential for male fertility through a genome-wide association study of non-obstructive azoospermia and RNA interference-mediated large-scale functional screening in Drosophila. Hum Mol Genet.

[23] Hu Z, Li Z, Yu J, Tong C, Lin Y, Guo X, Lu F, Dong J, Xia Y, Wen Y, Wu H, Li H, Zhu Y, Ping P, Chen X, Dai J (2014). Association analysis identifies new risk loci for non-obstructive azoospermia in Chinese men. Nat Commun.

[24] Turnbull C, Rapley EA, Seal S, Pernet D, Renwick A, Hughes D, Ricketts M, Linger R, Nsengimana J, Deloukas P, Huddart RA, Bishop DT, Easton DF, Stratton MR, Rahman N (2010). Variants near DMRT1, TERT and ATF7IP are associated with testicular germ cell cancer. Nat Genet.

[25] Kanetsky PA, Mitra N, Vardhanabhuti S, Vaughn DJ, Li M, Ciosek SL, Letrero R, D'Andrea K, Vaddi M, Doody DR, Weaver J, Chen C, Starr JR, Hakonarson H, Rader DJ, Godwin AK (2011). A second independent locus within DMRT1 is associated with testicular germ cell tumor susceptibility. Hum Mol Genet.

[26] Eisenberg ML, Betts P, Herder D, Lamb DJ, Lipshultz LI (2013). Increased risk of cancer among azoospermic men. Fertil Steril.

[27] Mancini M, Carmignani L, Gazzano G, Sagone P, Gadda F, Bosari S, Rocco F, Colpi GM (2007). High prevalence of testicular cancer in azoospermic men without spermatogenesis. Hum Reprod.

[28] Walsh TJ, Croughan MS, Schembri M, Chan JM, Turek PJ (2009). Increased risk of testicular germ cell cancer among infertile men. Arch Intern Med.

[29] Hu Z, Xia Y, Guo X, Dai J, Li H, Hu H, Jiang Y, Lu F, Wu Y, Yang X, Yao B, Lu C, Xiong C, Li Z, Gui Y, Liu J (2012). A genome-wide association study in Chinese men identifies three risk loci for non-obstructive azoospermia. Nat Genet.

[30] Insco ML, Leon A, Tam CH, McKearin DM, Fuller MT (2009). Accumulation of a differentiation regulator specifies transit amplifying division number in an adult stem cell lineage. Proc Natl Acad Sci U S A.

[31] Ahmad SM, Bhattacharyya P, Jeffries N, Gisselbrecht SS, Michelson AM (2015). Two Forkhead transcription factors regulate cardiac progenitor specification by controlling the expression of receptors of the fibroblast growth factor and Wnt signaling pathways. Development.

[32] Ahmad SM, Tansey TR, Busser BW, Nolte MT, Jeffries N, Gisselbrecht SS, Rusan NM, Michelson AM (2012). Two forkhead transcription factors regulate the division of cardiac progenitor cells by a Polo-dependent pathway. Dev Cell.

[33] Schuff M, Rossner A, Wacker SA, Donow C, Gessert S, Knochel W (2007). FoxN3 is required for craniofacial and eye development of Xenopus laevis. Dev Dyn.

[34] Samaan G, Yugo D, Rajagopalan S, Wall J, Donnell R, Goldowitz D, Gopalakrishnan R, Venkatachalam S (2010). Foxn3 is essential for craniofacial development in mice and a putative candidate involved in human congenital craniofacial defects. Biochem Biophys Res Commun.

[35] Nie X, Luukko K, Kettunen P (2006). BMP signalling in craniofacial development. Int J Dev Biol.

[36] Hamaratoglu F, Affolter M, Pyrowolakis G (2014). Dpp/BMP signaling in flies: from molecules to biology. Semin Cell Dev Biol.

[37] Restrepo S, Zartman JJ, Basler K (2014). Coordination of patterning and growth by the morphogen DPP. Curr Biol.

[38] Huot G, Vernier M, Bourdeau V, Doucet L, Saint-Germain E, Gaumont-Leclerc MF, Moro A, Ferbeyre G (2014). CHES1/FOXN3 regulates cell proliferation by repressing PIM2 and protein biosynthesis. Mol Biol Cell.

[39] Massague J (2012). TGFbeta signalling in context. Nat Rev Mol Cell Biol.

[40] Ren X, Sun J, Housden BE, Hu Y, Roesel C, Lin S, Liu LP, Yang Z, Mao D, Sun L, Wu Q, Ji JY, Xi J, Mohr SE, Xu J, Perrimon N (2013). Optimized gene editing technology for Drosophila melanogaster using germ line-specific Cas9. Proc Natl Acad Sci U S A.

[41] Chen D, McKearin D (2005). Gene circuitry controlling a stem cell niche. Curr Biol.

[42] Shang Y, Hu X, DiRenzo J, Lazar MA, Brown M (2000). Cofactor dynamics and sufficiency in estrogen receptor-regulated transcription. Cell.

[43] Tong C, Jiang J (2007). Using immunoprecipitation to study protein-protein interactions in the Hedgehog-signaling pathway. Methods Mol Biol.

