# Supplementary material for: CHES-1-like, the ortholog of a non-obstructive azoospermia-associated gene, blocks germline stem cell differentiation by upregulating Dpp expression in Drosophila testis
Source: Oncotarget. 2016 Jun 2;7(27):42303–13. doi: 10.18632/oncotarget.9789 (PMC5173136; doi:10.18632/oncotarget.9789)
Supplement: Supplementary file 1 [file oncotarget-07-42303-s001.pdf]

SUPPLEMENTARY FIGURES

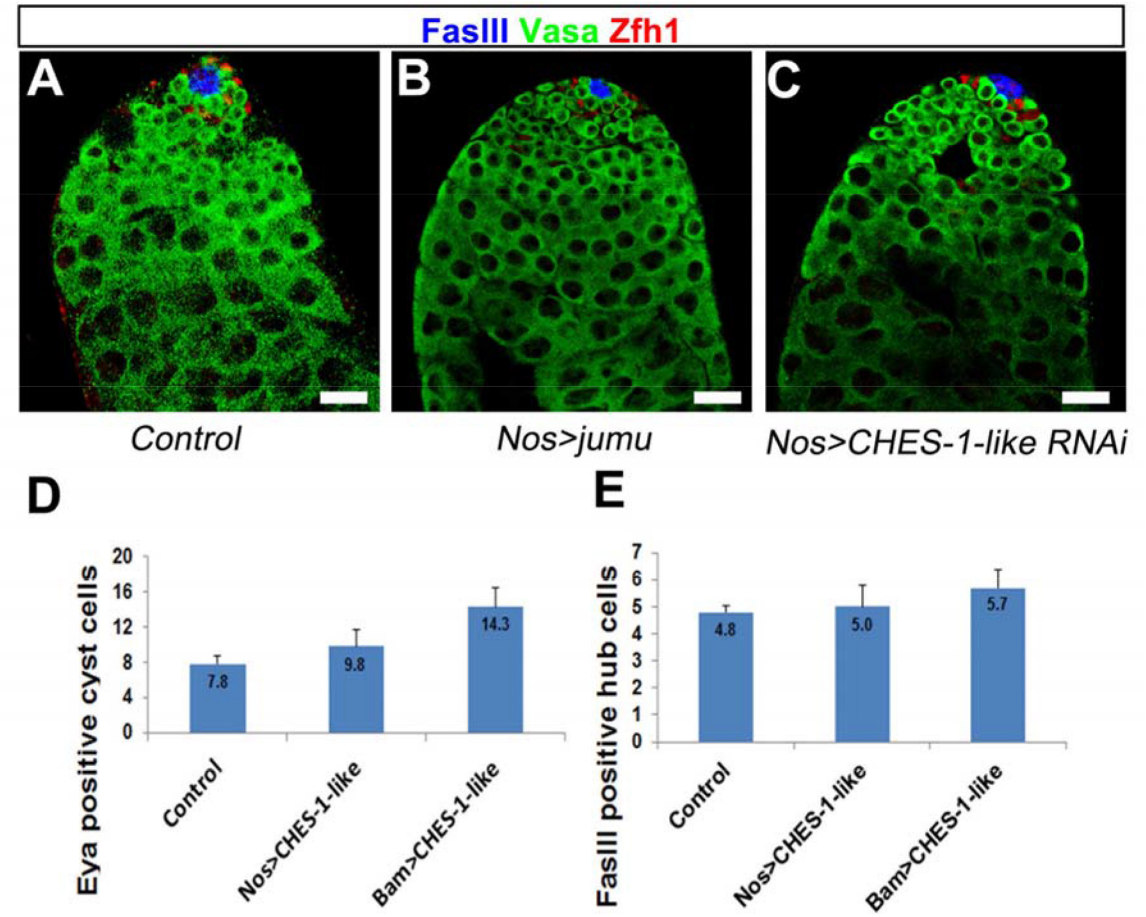

Supplementary Figure S1: A-C. Expressing *Jumu* or knocking down *CHES-1-like* in germ cells results morphological normal testes. Hub cells, germ cells, and cyst stem cells were labeled with FasIII, Vasa, and Zfh1 antibodies. Scale bars: 20 µM. **D, E.** The quantification of the somatic cell numbers in the testes with indicated genotypes.

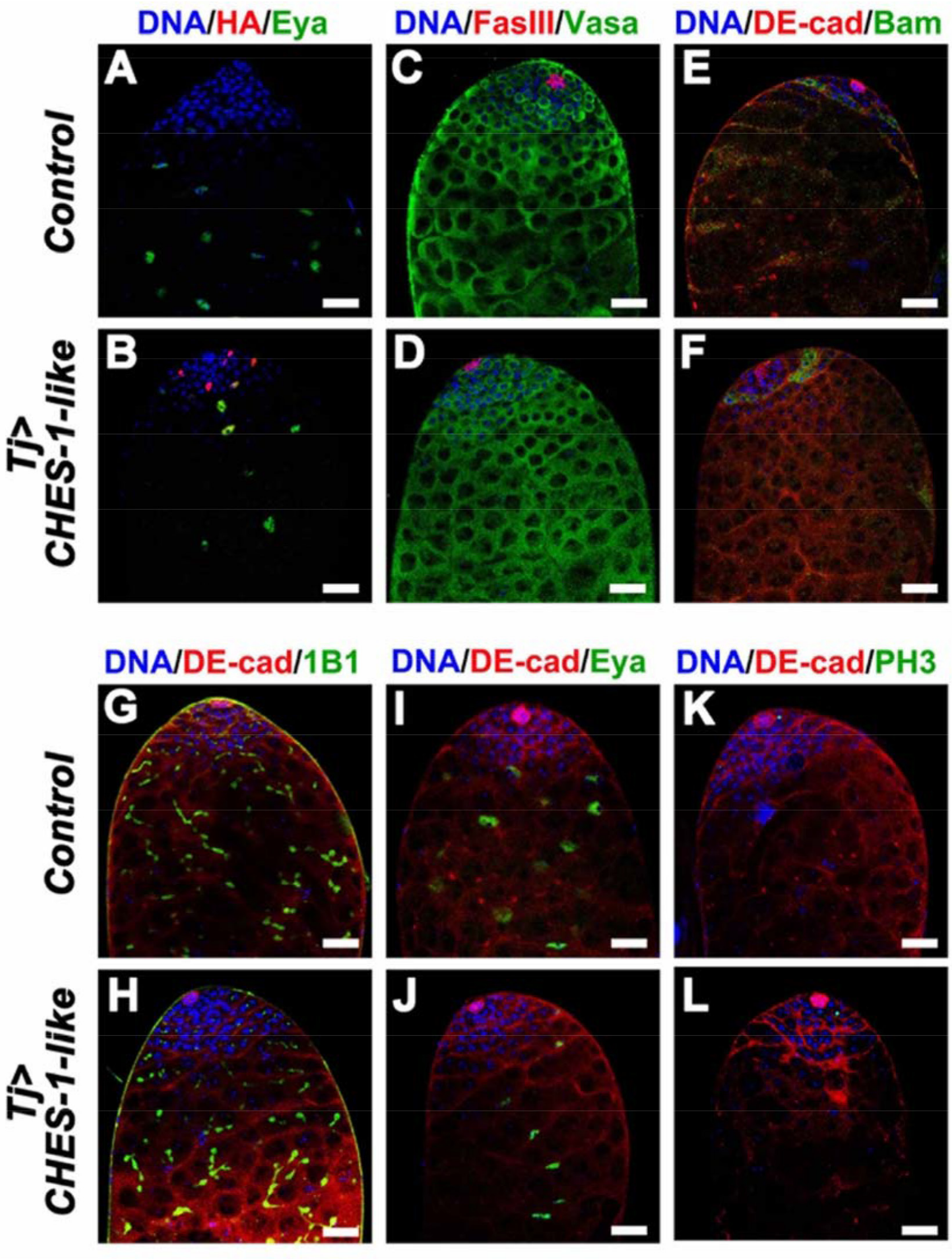

Supplementary Figure S2: Ectopic expressing CHES-1-like in cyst cells does not cause any structural defects in testes. DNA, HA tagged CHES-1-like, mature cyst cells, hub cells, germ cells, cyst cells, fusomes, Bam, and dividing cells were labeled with Hoechst 33342, HA, Eya, FasIII, Vasa, DE-cadherin, 1B1, Bam, and pH3 antibodies, respectively. Scale bars: 20 μM.

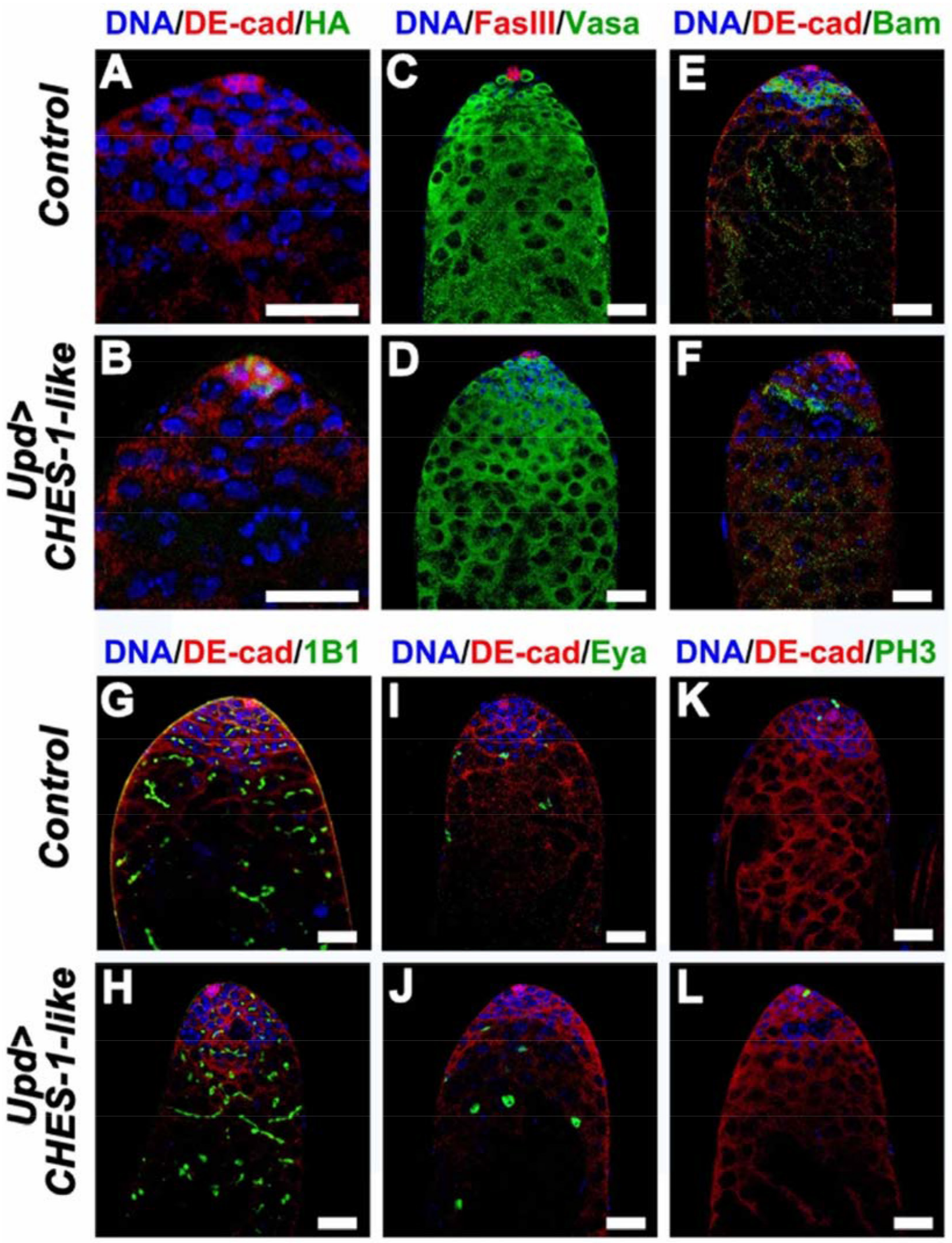

Supplementary Figure S3: Ectopic expressing CHES-1-like in hub cells does not cause testes defects. DNA, HA tagged CHES-1-like, mature cyst cells, hub cells, germ cells, cyst cells, fusomes, Bam, and dividing cells were labeled with Hoechst 33342, HA, Eya, FasIII, Vasa, DE-cadherin, 1B1, Bam, and PH3 antibodies, respectively. Scale bars: 20 μM.

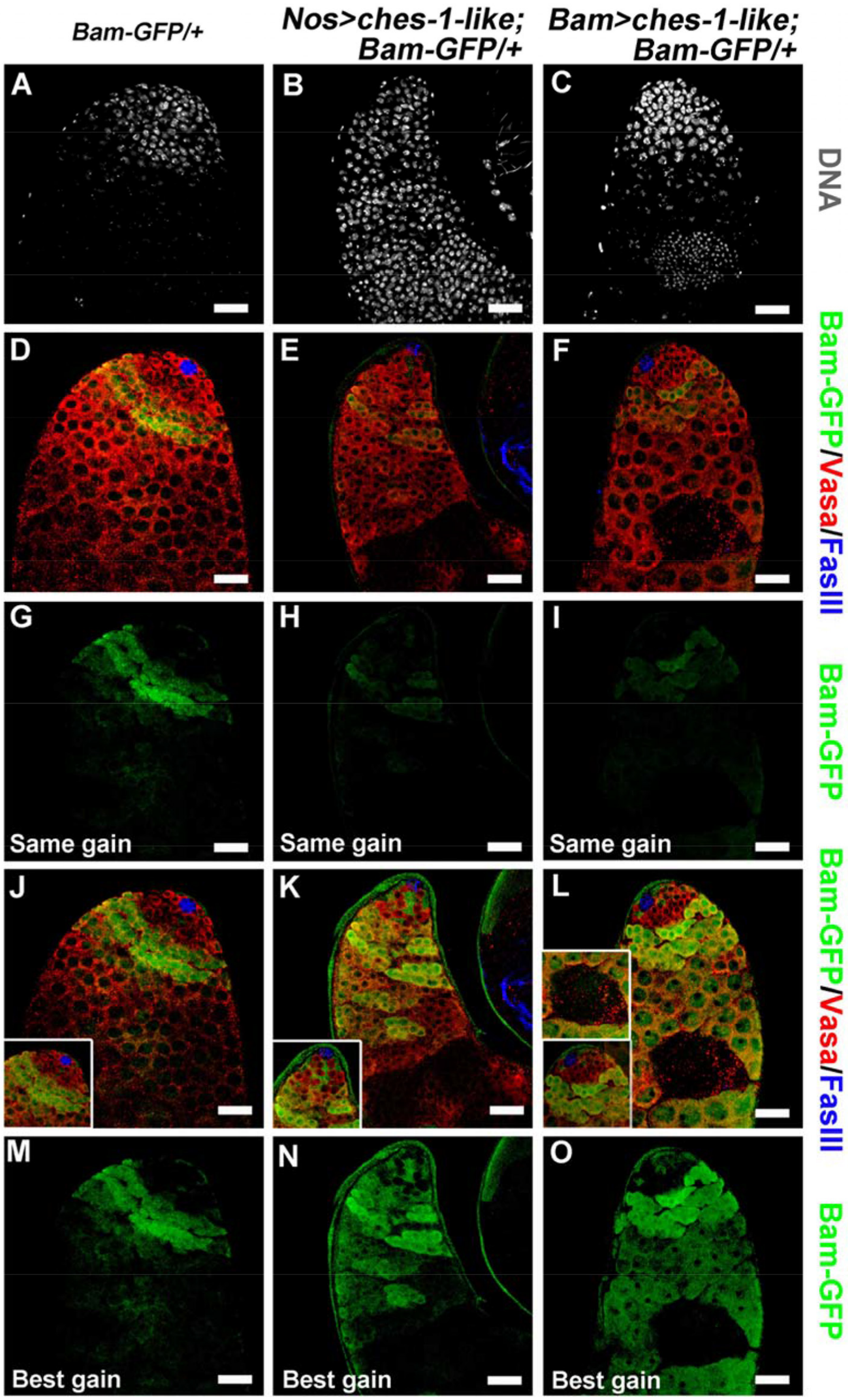

Supplementary Figure S4: Bam-GFP expression was reduced in the testes with germ cell ectopic expression of CHES-1-like. A-I. Bam-GFP expression levels are reduced in the testes with CHES-1-like ectopic expression in germ cells. The same gain were used to indicate the level of Bam-GFP expression. **J-O.** Using longer exposure time (best gain) to show the signals of GFP staining, expended GFP expression regions were observed in the testes with CHES-1-like overexpressed in germ cells. DNA (Grey), hub cells (Blue), and GFP (Green) were labeled with Hochest 33324, FasIII, and GFP antibodies. Scale bars: 20 µM.

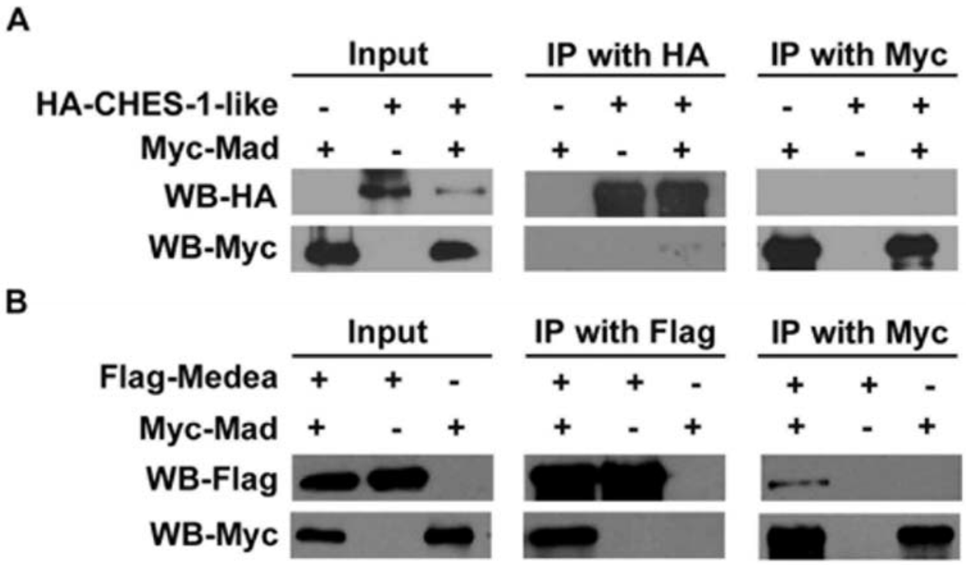

Supplementary Figure S5: There is no physical interaction between CHES-1-like and Mad. A. HA tagged CHES-1-like and Myc tagged Mad could not pull-down each other in an immunoprecipitation assay. **B.** As a positive control Flag tagged Medea and Myc-tagged Mad could pull-down each other.

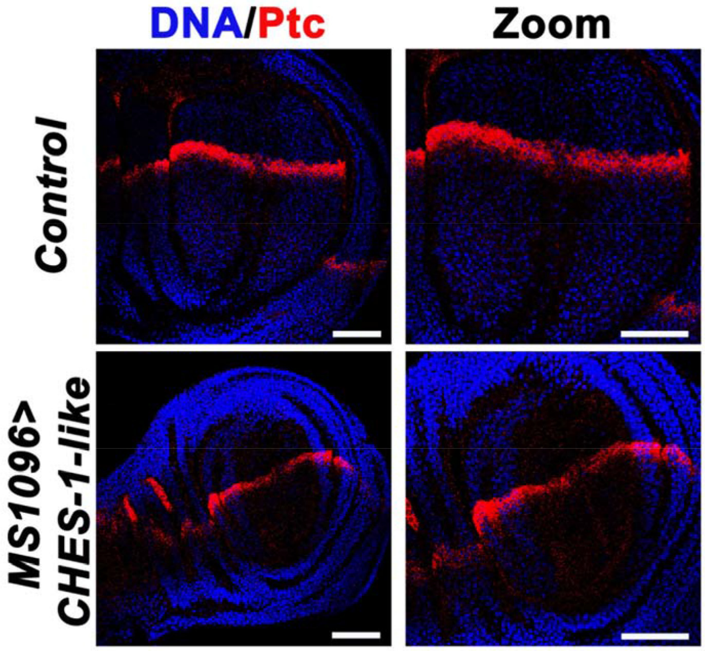

Supplementary Figure S6: Ptc expression patterns did not change in the CHES-1-like overexpression wing discs. DNA (Blue) and Ptc (Red) were labled with DAPI and anti-Ptc antibodies. Scale bars: 20 μM.
